# Early Massive Left Atrial Thrombosis Following Mitral Bioprosthetic Valve Replacement Due to Suboptimal Anticoagulation: A Case Report

**DOI:** 10.7759/cureus.92572

**Published:** 2025-09-17

**Authors:** Wanli Liu, Zhongkui Li, Chao Feng, Jun Liu

**Affiliations:** 1 Department of Cardiovascular Medicine, Guizhou Provincial People's Hospital, Guiyang, CHN

**Keywords:** atrial fibrillation, bioprosthetic mitral valve replacement, case report, left atrial thrombus, warfarin

## Abstract

Atrial thrombi are rare after mitral valve replacement. We report a case of early postoperative massive left atrial thrombus following bioprosthetic mitral valve replacement. The patient, a 61-year-old woman, underwent mitral valve replacement, tricuspid ring annuloplasty, and left atrial plication and was discharged after achieving the target international normalized ratio (INR) with oral warfarin. At the two-month follow-up, the imaging findings indicated a giant left atrial thrombus measuring 79 × 55 × 45 mm, with a subtherapeutic INR of 1.18. Anticoagulation therapy was intensified with warfarin and bridged with subcutaneous nadroparin. The thrombus decreased after three weeks and resolved completely by three months, and subsequent echocardiography showed valvular function was satisfactory. This case underscores the importance of strict INR monitoring for high-risk patients, highlighting that warfarin is an effective treatment option for left atrial thrombi following mitral valve replacement.

## Introduction

Antithrombotic regimens following cardiac valve replacement surgery are designed to minimize prosthetic leaflet thrombosis and subsequent systemic embolism [1]. Vitamin K antagonists (VKAs) have been proposed as the cornerstone of antithrombotic therapy for patients with heart valve replacement. Despite adequate anticoagulation therapy, patients remain at risk for thromboembolic events, and the management of such thrombi constitutes a significant therapeutic challenge. The reported annual incidence of prosthetic heart valve thrombosis ranges from 0.03% to 5.7% [2]. The majority of thrombotic events occur on the prosthetic valves, and isolated left atrial (LA) thrombus is an uncommon complication following mitral valve replacement. A previous study reported that the long-term incidence of LA thrombi following mitral valve replacement was 1.4%, with a minimum detection time of 17 months [3]. This case report describes the early formation of a massive LA thrombus, occurring just two months after bioprosthetic mitral valve replacement and attributed to suboptimal international normalized ratio (INR) control. We report this case to highlight the importance of careful INR monitoring and management strategies for early postoperative LA thrombus.

## Case presentation

**Figure 1:**
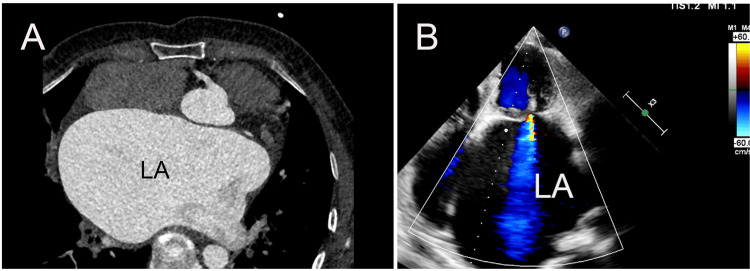
Cardiac CT and TTE showed a giant left atrium (LA). (A) Cardiac CT revealing a markedly enlarged LA compressing the adjacent cardiac chambers with a diameter of 75 mm. (B) TTE (apical four-chamber view) confirming the presence of a giant LA. CT: computed tomography; TTE: transthoracic echocardiography.

A 61-year-old woman, who presented with exertional palpitation, fatigue, and shortness of breath, was admitted to our hospital on June 26, 2023. There was no previous history of coronary heart disease, hypertension, or diabetes mellitus. She was diagnosed with atrial fibrillation by preoperative electrocardiography. Severe mitral stenosis and moderate-to-severe tricuspid insufficiency were determined by transthoracic echocardiography (TTE), with the mitral valve area of 0.3 cm^2^. Furthermore, the TTE and cardiac computed tomography (CT) showed a giant LA with a diameter of 75 mm (Figures 1A, 1B).

**Figure 2:**
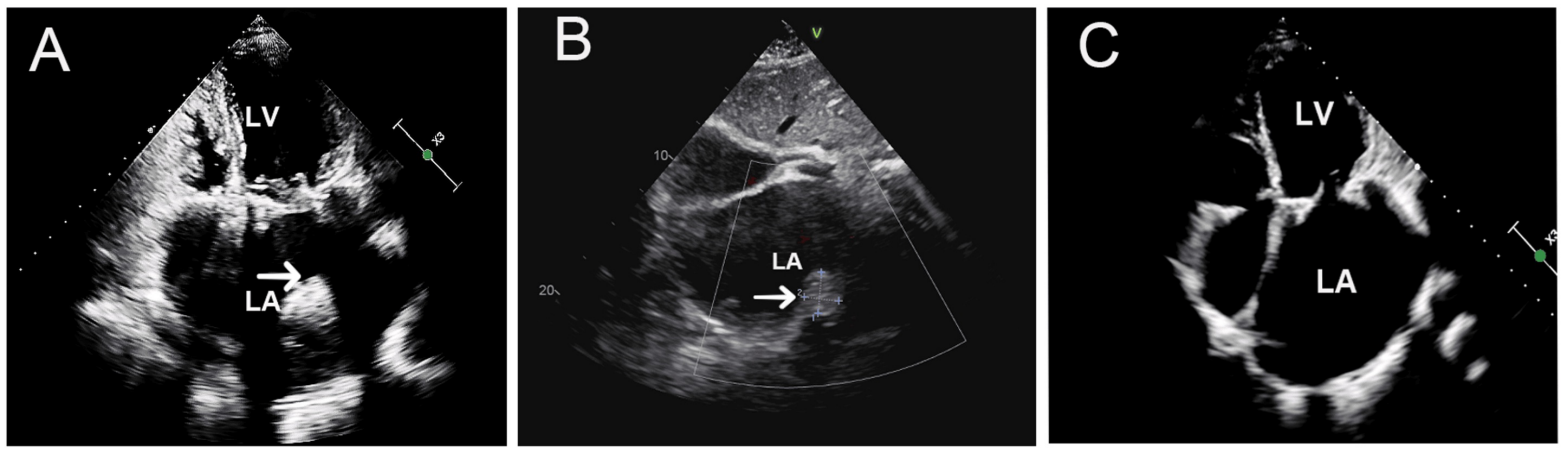
Transthoracic echocardiography showed the history of left atrial (LA) thrombus. Giant LA thrombus at baseline (arrow, A), with reduction after three weeks (arrow, B), and complete resolution after three months (C). LV: left ventricle.

After a full preoperative assessment, the patient underwent mitral valve replacement with a 27 mm bioprosthetic valve, tricuspid prosthetic ring annuloplasty, and LA plication. Oral warfarin anticoagulation was initiated on the first postoperative day, and the INR reached 2.64 on the 10th day postoperatively. A subsequent TTE showed that the prosthetic valve function was normal and the LA diameter reduced to 50 mm. Then, the patient was discharged three weeks after surgery with an INR of 1.9. She was advised to attend outpatient follow-up to adjust her warfarin dose and maintain her INR within the target range of 2.0-3.0. Two months after surgery, during regular outpatient follow-up, TTE demonstrated a LA thrombus (Figure 2A), with an INR of 1.18-well below the therapeutic range.

**Figure 3:**
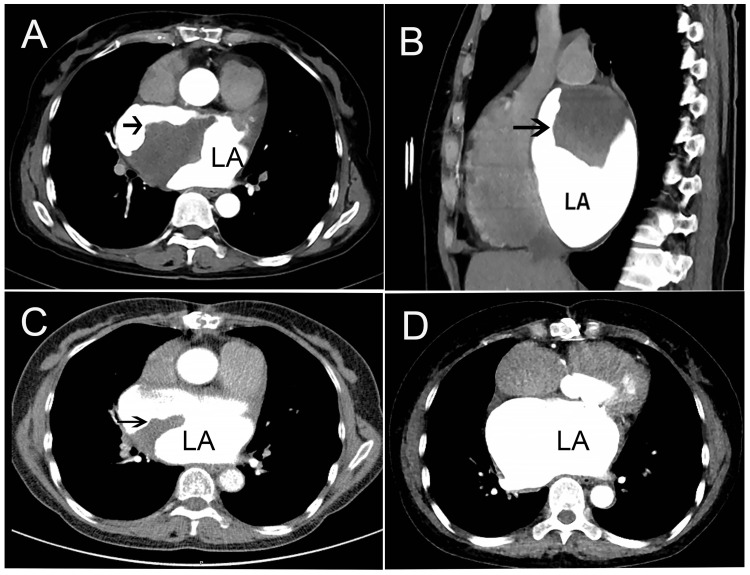
Cardiac CT demonstrating the resolution of a left atrial (LA) thrombus. Transverse (A) and sagittal (B) views showing a thrombus within the LA (arrows), with an irregular contour, measuring 79 × 55 × 45 mm. (C) Follow-up CT at 3 weeks demonstrated a marked reduction in thrombus size (arrow). (D) Follow-up CT at 3 months confirmed complete resolution of the thrombus. CT: computed tomography.

She was readmitted to the hospital, and cardiac CT showed a large irregular thrombus in the LA with a lesion size of 79 × 55 × 45 mm (Figures 3A, 3B).

**Figure 4:**
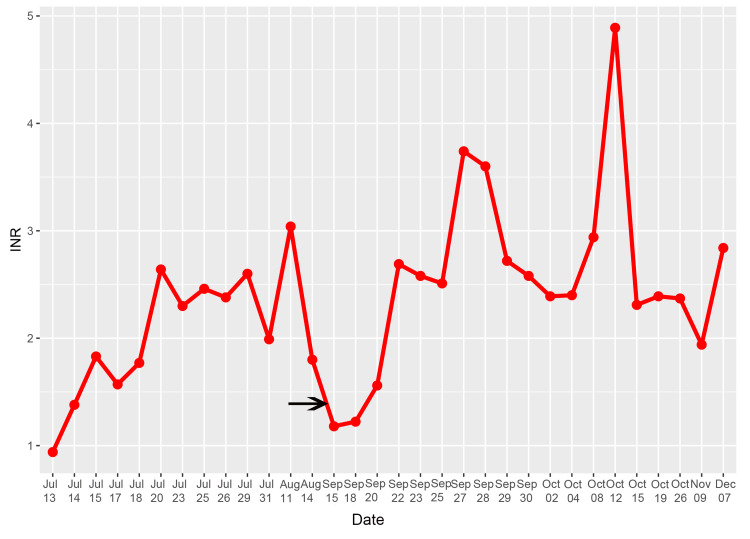
Fluctuation of INR values. The patient’s INR was therapeutic at discharge. A subsequent drop to subtherapeutic levels (~1.18, marked by the arrow) preceded left atrial thrombus formation. The patient was readmitted and received therapeutic-dose LMWH bridging while warfarin was adjusted. INR returned to the target range after approximately one week, and oral warfarin alone was continued to maintain INR between 2.0 and 3.0. The thrombus subsequently reduced in size and resolved. LMWH: low-molecular-weight heparin; INR: international normalized ratio.

The patient was treated with therapeutically subcutaneous nadroparin calcium, and the oral warfarin dose was adjusted. When the INR reached the therapeutic range after one week, nadroparin calcium was discontinued, and the treatment strategy was to continue oral warfarin to maintain the INR of 2.0 to 3.0. Three weeks later, cardiac CT (Figure 3C) and TTE (Figure 2B) both revealed that the size of the thrombus was reduced, and the size on cardiac CT was 41 mm × 32 mm (Figure 3C). Then, she was discharged and instructed to continue oral warfarin, aiming to maintain her INR within the target therapeutic range. After discharge, her INR showed some variability, yet consistently stayed within the therapeutic range (Figure 4).

Approximately three months after the thrombus was identified, a repeated CT scan (Figure 3D) and TTE (Figure 2C) showed that the thrombus in the LA was absent. There were no thrombus-related complications, and the patient was continuously treated with oral warfarin. Echocardiographic evaluation after one year demonstrated the absence of thrombus recurrence, and the prosthetic valve function was normal.

## Discussion

We presented a rare case of a giant LA thrombus after mitral valve replacement associated with some risk factors. Atrial thrombi are rare after mitral valve replacement, and Chen et al. reported the long-term rate was 1.4% [3]. VKAs are recommended lifelong for patients undergoing mechanical prosthetic valve replacement surgery and indicated within three months following surgical replacement of biological prosthetic valves [1]. The aim of anticoagulant therapy for patients with prosthetic valves is to reduce the incidence of thromboembolic complications, including valve thrombosis or systemic embolism. The risk factors consist of atrial fibrillation, previous thromboembolism, a hypercoagulable state, and impaired LV dysfunction [4]. A previous study has demonstrated that enlarged LA diameter (>55 mm), elevated mitral valve pressure gradient (MVPG >6 mmHg), and reduced left ventricular (LV) function (LVEF <50%) are independent risk factors for LA thrombi after mitral valve replacement [3]. In summary, the mitral bioprosthetic replacement patient reported in our case exhibited two established thrombogenic risk factors: atrial fibrillation and a giant LA (LA diameter of 75 mm). Therefore, rigorous postoperative anticoagulation therapy is imperative for this patient.

Inadequate anticoagulation is another risk factor for thromboembolic events. Warfarin, the major clinically used oral VKA, requires monitoring and dose adjustment guided by INR. In accordance with the 2020 American College of Cardiology (ACC)/American Heart Association (AHA) guideline for the management of patients with valvular heart disease (Class IIa), sustained anticoagulation with a VKA to maintain an INR of 2.0 to 3.0 is recommended for patients undergoing prosthetic valve replacement with atrial fibrillation [5]. Fluctuation in INR is a strong independent predictor of adverse events after valve replacement. It has been reported that inadequate anticoagulation is a risk factor for mechanical valve obstruction [6]. At our institution, patients on warfarin are advised to attend monthly outpatient visits. The patient’s INR was therapeutic at discharge (1.9), which fell to 1.18 over one month, during which a thrombus developed. The patient denied any history of using oral herbal medications or other drugs in the past month, so the cause of suboptimal INR was not clear. Meanwhile, the LA thrombus was detected. Therefore, we concluded that the LA thrombus was associated with suboptimal INR control.

Current therapeutic consensus for LA thrombi after mitral valve replacement lacks robust clinical validation, resulting from epidemiological rarity. Surgery and oral warfarin have been reported for the treatment of LA thrombi after mitral valve surgery. Marschall et al. reported a case of performing a percutaneous LA appendage closure to prevent the recurrence of LA appendage thrombus in a patient with recurrent embolisms after prosthetic heart valve replacement [7]. However, this technique can only be used in patients with LA appendage thrombus. For patients with LA ball thrombus after mitral valve surgery, emergency surgery was considered the first-line treatment because there is a risk of sudden cardiac death [8,9]. Rosa et al. presented a case in which an LA thrombus was detected after warfarin was discontinued for suspected hepatotoxicity, and TTE at one month post-warfarin reinitiation confirmed the resolution of the previously identified LA thrombus [10]. Despite established therapeutic strategies for LA thrombus post-mitral valve surgery, robust clinical treatment evidence remains absent for massive thrombi occurring in the early postoperative phase following bioprosthetic mitral valve replacement.

Given that the thrombus was detected two months after surgery, this means that the emergency surgery and fibrinolysis were not the first choice for our case, considering the bleeding risk. Furthermore, the thrombus did not affect the prosthetic valve function, and the patient was hemodynamically stable. Hence, we increased the dose of warfarin to reach the INR goal. At the same time, subcutaneous low-molecular-weight heparin was applied for bridging. The LA thrombus demonstrated a significant reduction following the achievement of therapeutic INR at three weeks, with essential resolution observed by three months. She was advised to continue oral warfarin anticoagulation therapy. At the time of this report, the patient maintained satisfactory valvular function without experiencing thrombotic complications.

## Conclusions

In conclusion, the development of massive LA thrombi during the early postoperative phase following mitral valve replacement is an extremely rare clinical entity. Careful INR monitoring to maintain the target range with warfarin anticoagulation after mitral valve replacement is critically important, particularly in patients with risk factors such as atrial fibrillation or a significantly enlarged LA. Optimizing anticoagulation using warfarin is an alternative treatment for patients with LA thrombi after bioprosthetic mitral valve replacement.
